# Genome-Wide Identification of the *MPK* Gene Family and Expression Analysis under Low-Temperature Stress in the Banana

**DOI:** 10.3390/plants12162926

**Published:** 2023-08-12

**Authors:** Zhengyang Fan, Bianbian Zhao, Ruilian Lai, Huan Wu, Liang Jia, Xiaobing Zhao, Jie Luo, Yuji Huang, Yukun Chen, Yuling Lin, Zhongxiong Lai

**Affiliations:** 1 Institute of Horticultural Biotechnology, Fujian Agriculture and Forestry University, Fuzhou 350002, China; ffzzyy789@163.com (Z.F.); zbb@fafu.edu.cn (B.Z.); lairl0618@163.com (R.L.); wuhuan980422@163.com (H.W.); kieraj@163.com (L.J.); zxb1344@126.com (X.Z.); luojie1998s@163.com (J.L.); yjhuang2004@163.com (Y.H.); cyk68@163.com (Y.C.); buliang84@163.com (Y.L.); 2 Fruit Research Institute, Fujian Academy of Agricultural Sciences, Fuzhou 350013, China

**Keywords:** banana, MAPK, low temperature stress, miR172

## Abstract

Mitogen-activated protein kinases (MAPKs and MPKs) are important in the process of resisting plant stress. In this study, 21, 12, 18, 16, and 10 *MPKs* were identified from *Musa acuminata*, *Musa balbisiana*, *Musa itinerans*, *Musa schizocarpa*, and *Musa textilis*, respectively. These *MPKs* were divided into Group A, B, C, and D. Phylogenetic analysis revealed that this difference in number was due to the gene shrinkage of the Group B subfamily of *Musa balbisiana* and *Musa textilis*. KEGG annotations revealed that K14512, which is involved in plant hormone signal transduction and the plant–pathogen interaction, was the most conserved pathway of the *MPKs*. The results of promoter cis-acting element prediction and *foc*TR4 (*Fusarium oxysporum* f. sp. *cubense* tropical race 4) transcriptome expression analysis preliminarily confirmed that *MPKs* were relevant to plant hormone and biotic stress, respectively. The expression of *MPKs* in Group A was significantly upregulated at 4 °C, and dramatically, the *MPKs* in the root were affected by low temperature. miR172, miR319, miR395, miR398, and miR399 may be the miRNAs that regulate *MPKs* during low-temperature stress, with miR172 being the most critical. miRNA prediction and qRT-PCR results indicated that miR172 may negatively regulate *MPKs*. Therefore, we deduced that *MPKs* might coordinate with miR172 to participate in the process of the resistance to low-temperature stress in the roots of the banana. This study will provide a theoretical basis for further analysis of the mechanism of *MPKs* under low-temperature stress of bananas, and this study could be applied to molecular breeding of bananas in the future.

## 1. Introduction

MAPK cascades are conserved in eukaryotes, which play an important role in plant development [1,2,3] and the stress-response mechanism [4,5,6]. Upstream signals of MPKs include mitogen-activated protein kinase kinase (MAPKK, MKK, and MEK) and mitogen-activated protein kinase kinase kinase (MAPKKK, MKKK, and MEKK) [7]. In general, MPKs relate to a variety of kinases with similar functions, which diversify the signal transduction pathways in plants to meet the needs of plant growth and development in complex environments. MKKKs, MKKs, and MPKs are phosphorylated in the signal transduction pathways. MPKs are activated and transferred to the nucleus or cytoplasm [8], where they then promote the expression of transcription factors or phosphorylate downstream substrates [9]. Eventually, these phosphorylation processes enable plant cells to respond to external signals.

Bananas are widely cultivated in tropical and subtropical regions as one of the main food crops in the world. However, bananas are sensitive to low temperatures, which indicates that high temperature and humidity conditions are better for their growth. Although researchers have conducted a large number of studies, the molecular mechanism underlying the cold resistance of bananas has not been completely understood [10,11,12]. AtMPK3/6 are negative regulators of the AtCBF-dependent cold response network in *Arabidopsis*. The AtICE1 protein has a capacity of being phosphorylated by AtMPK3/6 to inhibit downstream AtCBF expression, thereby affecting the tolerance of plants to low-temperature conditions [13]. Cascades of AtMEKK1-AtMKK2-AtMPK4 and AtMKK4/5-AtMPK3/6 play important roles in cold stress, in which a low temperature rapidly induces the expression of *AtMPK3/6* [14]. As a result of being phosphorylated by AtMPK3/6, the AtICE1 protein will not be able to regulate AtCBF effectively as a result. Eventually, the cold resistance of *Arabidopsis thaliana* is reduced, thereby showing how a negative regulatory network works [15]. Furthermore, the cascade of AtMEKK1-AtMKK2-AtMPK4 inhibits the phosphorylation of AtICE1 by AtMPK3/6, improving the stability of AtICE1, which will enhance the cold resistance of the plant as a result [16]. Interestingly, compared with AtMPK3/6, OsMPK3, which is present in rice, plays an opposite role under cold stress. Rice responds to cold stress through the OsMPK3-OsICE1-OsTPP1 pathway. OsICE1 activates OsTPP1 to promote the accumulation of trehalose in rice to resist low-temperature stress [17]. Therefore, it is significant for scientific research and agricultural production to analyze the mechanism of banana *MPKs* under cold stress. MAPK cascades have the capacity of improving the cold resistance of waxy corn [18]. Mel enhanced cold tolerance in the winter turnip rape crop (*Brassica rapa* L.), which depends on *MPK3/6* [19]. *SlMPK1/2* can interact with *SlBBX13* to improve cold tolerance in the tomato plant [20]. The COG1–OsSERL2 complex can promote the activation of OsMPK3 under low temperatures, thereby enhancing the cold resistance of japonica rice [21]. Previous studies have identified 25 members of the *MPK* gene family in the first version of the genome of *Musa acuminata* [22]; however, the differences and associations between the *MPKs* of different banana genomes have not been analyzed as of yet.

In this study, we analyzed the *MPKs* of five representative bananas. The *MPK* gene family was identified as *Musa acuminata*, *Musa balbisiana*, *Musa itinerans*, *Musa schizocarpa*, and *Musa textilis*. We then screened out a gene that is very critical to cold stress. In addition, the relationship between miR172 and *MPKs* was also researched. This study will provide the theoretical basis for further analysis of the mechanism of *MPKs* under cold stress in bananas, and this study may be applied to the molecular breeding of bananas in the future.

## 2. Results

### 2.1. Identification of the MPK Gene Family in Bananas

Based on the banana genome database, 21, 12, 18, 16, and 10 *MPK* family members were identified in *Musa acuminata*, *Musa balbisiana*, *Musa itinerans*, *Musa schizocarpa*, and *Musa textilis,* respectively. According to the location of these genes on chromosomes, they were named *MaMPK1-21*, *MbMPK1-12*, *MiMPK1-18*, *MsMPK1-16,* and *MtMPK1-10*, respectively. The number of amino acids, molecular weight, theoretical pI, instability index, and aliphatic index of the *MPKs* were between 366–1155, 41.68–130.81 KDa, 5.34–9.73, 28.07–51.77, and 99.1–78.41, respectively. GRAVY results revealed that these *MPKs* were hydrophilic proteins (Table S1).

### 2.2. Phylogenetic Analysis of the MPK Gene Family

**Figure 1:**
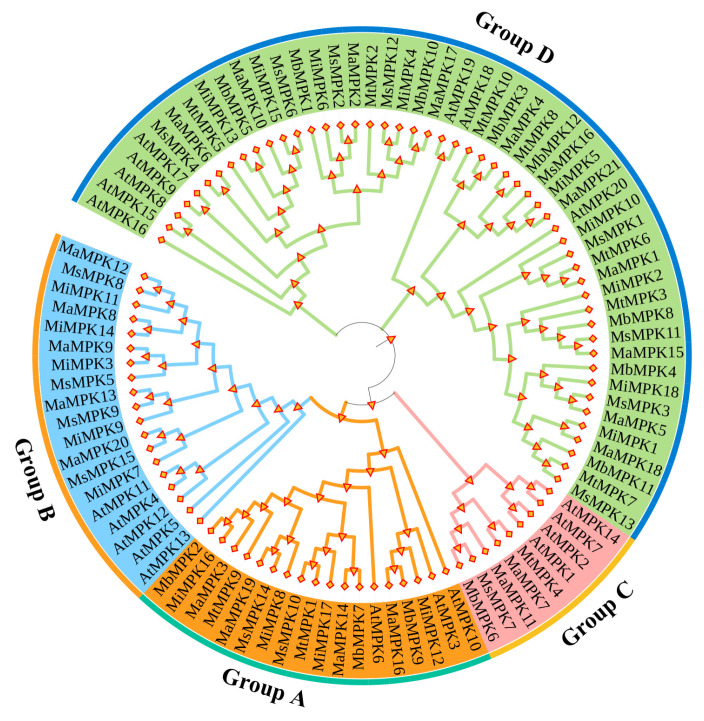
Phylogenetic analysis of the *MPK* gene family in *Arabidopsis thaliana* (At), *Musa acuminata* (Ma), *Musa balbisiana* (Mb), *Musa itinerans* (Mi), *Musa schizocarpa* (Ms), and *Musa textilis* (Mt).

To explore the relationship of these *MPKs* in bananas, MEGA-X was used to analyze the amino acid sequences and to construct the neighbor-joining (NJ) phylogenetic tree. According to the classification of *AtMPKs*, the phylogenetic tree was divided into groups A, B, C, and D (Figure 1).

**Figure 2:**
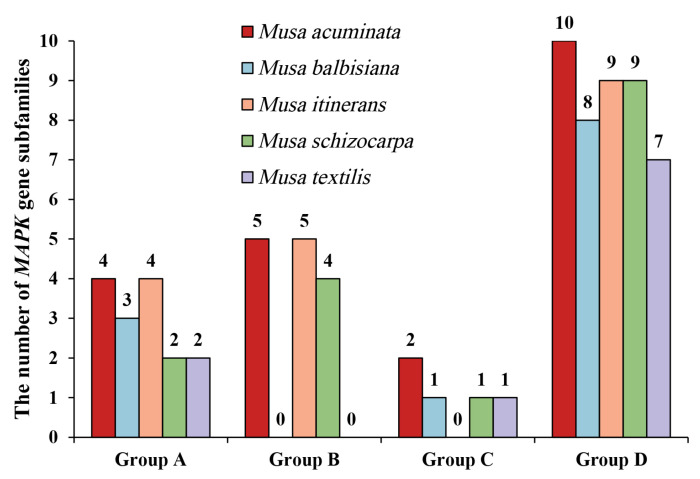
The number of *MPK* subfamily members.

Due to the difference in the number of *MPKs* in genomes, the number of subfamilies was counted according to the phylogenetic tree (Figure 2). It was clear that the quantity of *MPKs* was approximately equal in Group A, C, and D. Notably, no family members of *Musa balbisiana* and *Musa itinerans* were clustered in Group B. Therefore, the main reason for the difference in the amount of *MPKs* in bananas was the gene shrinkage of the Group B subfamily of *Musa balbisiana* and *Musa itinerans*.

### 2.3. Colinearity Analysis of the MPK Gene Family

**Figure 3:**
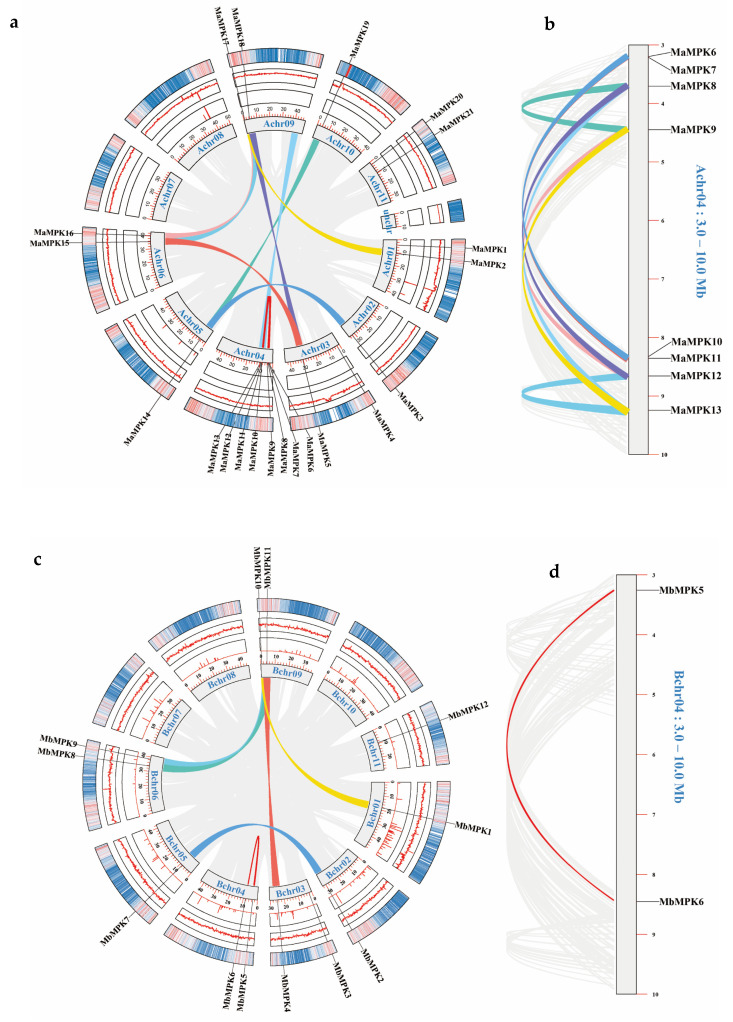

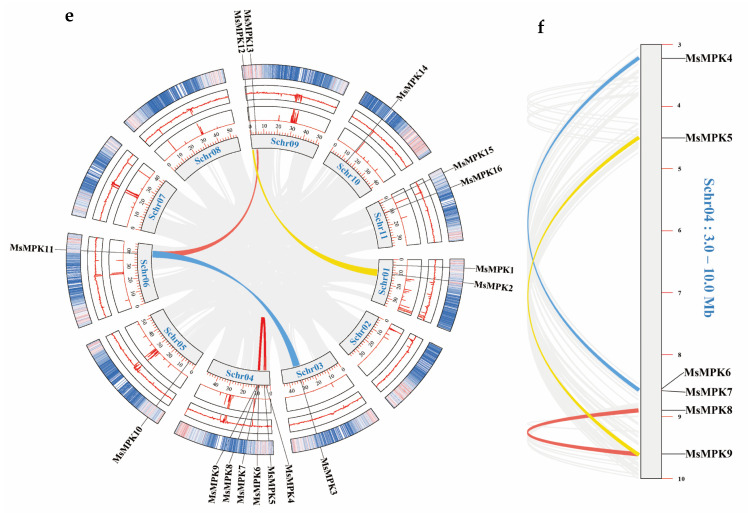
Collinearity analysis of the *MPK* gene family in bananas. The rings from the outside to the inside represent gene density, GC content, unknown base content, and pseudochromosomes. (**a**) Collinearity analysis of *Musa acuminata*. The colorful lines indicate the collinearity between the *MaMPK*s. (**b**) The 3–10 MB region of the Chr04 of *Musa acuminata.* (**c**) Collinearity analysis of *Musa balbisiana*. The colorful lines indicate the collinearity between the *MbMPK*s. (**d**) The 3–10 MB region of the Chr04 of *Musa balbisiana*. (**e**) Collinearity analysis of *Musa schizocarpa.* The colorful lines indicate the collinearity between the *MsMPK*s. (**f**) The 3–10 MB region of the Chr04 of *Musa schizocarpa*.

Collinear analysis of the whole genome was used to understand gene duplication events of the *MPK* gene family. Meanwhile, unknown base content, GC content, and gene density data were supplemented as references. Unfortunately, the *Musa itinerans* and *Musa textilis* genomes were not assembled to the pseudochromosome level, meaning only *Musa acuminata*, *Musa balbisiana,* and *Musa schizocarpa* were analyzed (Figure 3). There were eight, six, and three segmental duplications and eight, zero, and three tandem duplications observed between the *MPK* family of *Musa acuminata*, *Musa balbisiana,* and *Musa schizocarpa,* respectively (Figure 3a,c,e). Interestingly, the 3–10 MB regions of Chr04 in *Musa acuminata* and *Musa schizocarpa* were discovered through eight and three times of segmental duplication, respectively (Figure 3b,f), but were not found in this region of *Musa balbisiana* (Figure 3d). The interspecific features of *MbMPKs* may come into being from long-term evolution and natural selection. These gene duplication events may refer to the functional differentiation and expansion of the *MPK* gene family in bananas.

**Figure 4:**
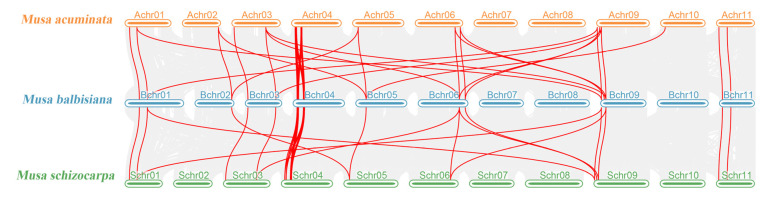
Collinearity analysis of the *MPK* gene family in *Musa acuminata*, *Musa balbisiana,* and *Musa schizocarpa*. The red lines mark the *MPK* gene pairs in bananas.

The results of collinearity analysis among *Musa acuminata*, *Musa balbisiana,* and *Musa schizocarpa* reveal that the positions of these *MPKs* on the chromosomes were also very similar (Figure 4), indicating that *MPKs* are quite conserved in bananas. Similarly, *MPKs* also have close genetic relationships in *Musa acuminata*, *Musa balbisiana,* and *Musa schizocarpa*. These results suggested that *MPKs* located in 3–10 MB of Chr04 may have evolved from the same gene.

### 2.4. KEGG Annotation Analysis of the MPK Gene Family

**Figure 5:**
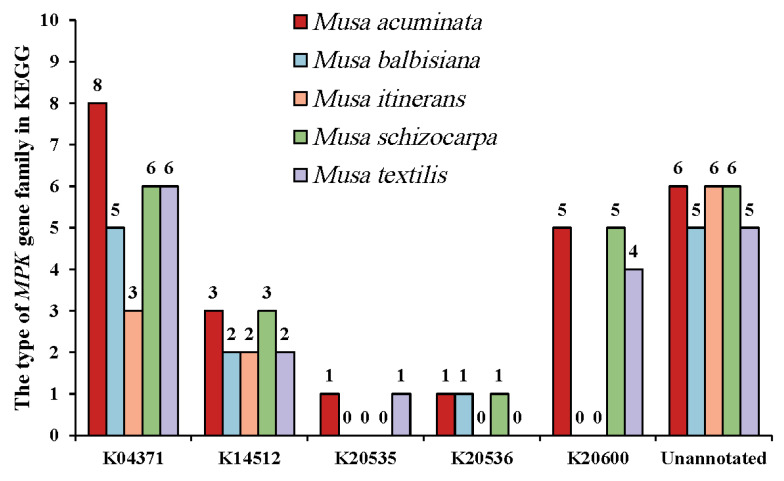
The type of *MPK* gene family in KEGG. One protein sequence may correspond to multiple entries.

To further verify the functional similarities and differences of the *MPK* gene family, functional annotations were made on their protein sequences (as shown in Figure 5). Most of the *MPKs* were annotated to K04371, K14512, K20535, K20536, and K20600, and others were not annotated. It is noteworthy that K14512 is the most conserved in bananas and existed in all species. K14512 is divided into three pathways, which plays an important role in plant growth and development. These pathways contains plant hormone signal transduction (map04075), plant–pathogen interaction (map04626), and MAPK signaling pathway–plant (map04016).

### 2.5. Prediction Analysis of the MPKs Promoter Cis-Acting Elements

**Figure 6:**
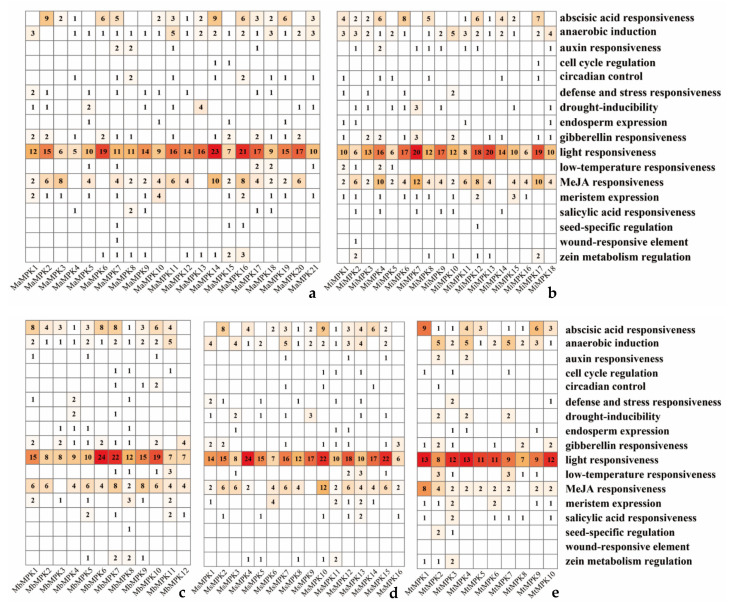
Prediction analysis of *MPKs* promoter cis-acting elements in *Musa acuminata* (**a**), *Musa itinerans* (**b**), *Musa balbisiana* (**c**), *Musa schizocarpa* (**d**), and *Musa textilis* (**e**). The color depth of the square represents the number of cis-acting elements.

The results of promoter analysis showed that *MPKs* promoter cis-acting elements were related to plant hormones, the stress response, biological clocks, and plant development (Figure 6). In terms of plant hormones, it mainly contained abscisic acid, auxin, gibberellin, methyl jasmonate, and salicylic acid response elements, which, to some extent, confirmed the conclusion that *MPK* participated in plant hormone signal transduction pathways in KEGG. The stress response is mainly involved in anaerobic induction, stress defense, drought inducibility, low-temperature responsiveness, and wound responsiveness. Elements of cell cycle regulation and circadian control response are related to the biological clock regulation system. Finally, endosperm expression, light responsiveness, meristem expression, seed-specific regulation, and zein metabolism regulation elements are involved in plant development.

Overall, the most responsive element in the promoters was light responsiveness, while the least responsive element was wound responsiveness. This result suggested that *MPKs* may mainly rely on light, abscisic acid, and the MeJA metabolic pathways, which was found to be similar across different species of bananas. However, wound responsiveness was only found in the promoters of *MaMPK7* and *MiMPK2* (Figure 6a,b), but not in the others (Figure 6c–e). Seed-specific regulation did not exist in *Musa schizocarpa* (Figure 6d) but was found in all the others. In summary, the promoters of *MPKs* are both conserved and specific. A variety of promoter cis-acting elements could provide the *MPKs* with more functions.

### 2.6. Prediction of miRNA Regulation on MaMPKs

**Figure 7:**
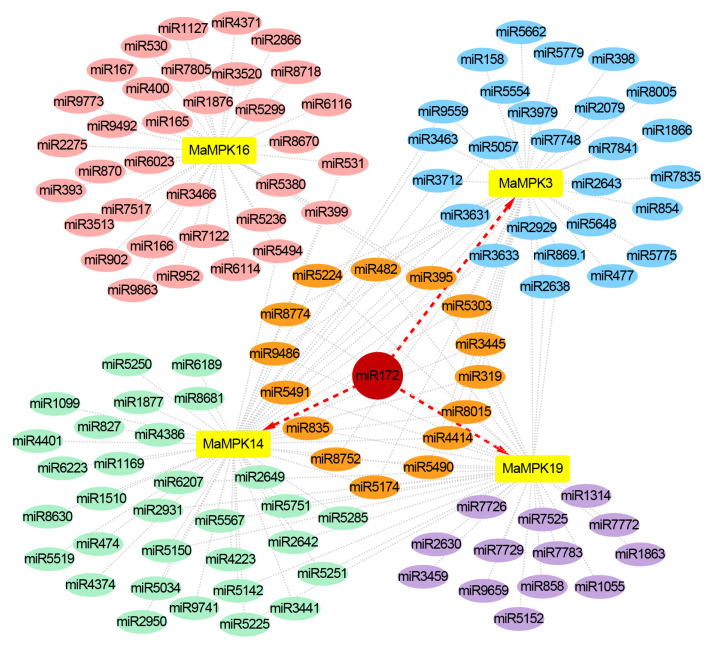
The interaction network between miRNAs and *MaMPKs*. The yellow rectangles represent genes, and the other ellipses represent miRNAs. The orange and red ellipses represent miRNAs that regulate three genes simultaneously. The pink, blue, green, and purple ellipses represent miRNAs that regulate one gene. This interaction network was visualized using Cytoscape.

**Figure 8:**
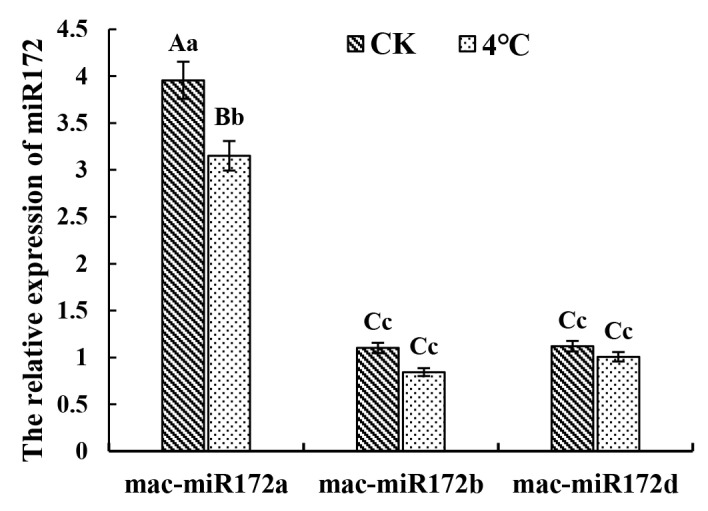
The expression of miR172 under low-temperature stress. Uppercase and lowercase letters indicate a significant difference at *p* < 0.01 and 0.05.

A total of 119 types of miRNAs regulating the *MPKs* were predicted by psRNATarge (Figure 7). Interestingly, sixteen miRNAs were found to regulate three genes simultaneously. *MaMPK3*, *MaMPK14,* and *MaMPK16* were regulated by miR5303; *MaMPK14*, *MaMPK16,* and *MaMPK19* were regulated by miR395; *MaMPK3*, *MaMPK14,* and *MaMPK19* were regulated by the other 14 miRNAs. Notably, the regulation of miR172 on *MaMPK3*, *MaMPK14,* and *MaMPK19* was found to be the most significant. Therefore, three miR172 family members were screened from the banana miRNA database for expression analysis under low-temperature stress (Figure 8). The results showed that mac-miR172a was significantly downregulated, while mac-miR172b and mac-miR172d were hardly affected by the low temperature. The expression level of mac-miR172a was about 4-fold higher than that of mac-miR172b and mac-miR172d at 28 °C. The expression of mac-miR172a was downregulated at 4 °C, indicating that mac-miR172a plays a major role in low-temperature stress. These results indicate that miR172 has a functional redundancy in bananas. In addition, miRNAs associated with cold stress, such as miR319, miR395, miR398, and miR399, were also predicted. This suggested that the regulatory mechanisms of miRNAs on *MaMPKs* were diverse.

### 2.7. Expression Patterns of MPKs under Biotic and Abiotic Stress

**Figure 9:**
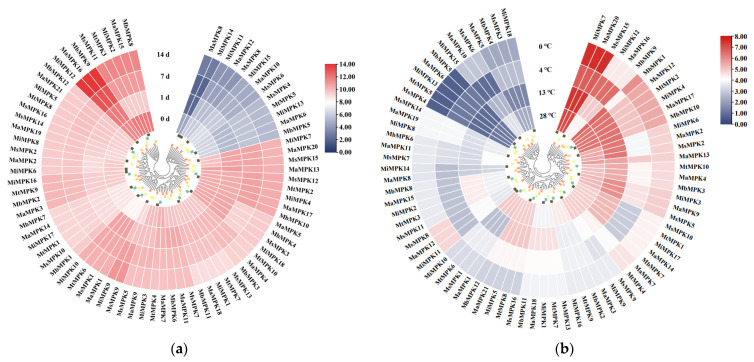
Expression patterns of *MPKs* under biotic and abiotic stress. (**a**) *Fusarium oxysporum* f. sp. *cubense* tropical race 4 (*Foc*TR4)-infected bananas at 0, 1, 4, and 7 days. (**b**) Bananas were treated at 0 °C, 4 °C, 13 °C, and 28 °C for 24 h. Red represents high expression and dark blue represents low expression.

The transcriptome FPKM data were used to analyze the expression patterns of *MPKs*, including pathogen stress and low-temperature stress (Figure 9). Thirteen genes were identified with low expression levels after 0, 1, 4, and 7 days of *Fusarium oxysporum* f. sp. *cubense* tropical race 4 (*Foc*TR4) infection (Figure 9a), and their expression decreased with the extension of the infection time. The expression levels of the other *MPKs* were found to be higher than these 13 genes. Among them, the expression of *MaMPK8* and *MiMPK14* belonging to the Group B subfamily decreased most significantly. It is noteworthy that most of these *MPKs* are highly expressed. The expression of *MiMPK12*, *MaMPK16*, and *MbMPK9* increased most significantly following pathogen induction. *MsMPK11*, *MtMPK3*, *MiMPK2*, *MaMPK15*, and *MbMPK8* were downregulated from 0–1 d and upregulated from 1–14d. These results indicate that most *MPKs* can respond to *foc*TR4, especially *MiMPK12*, *MaMPK16*, and *MbMPK9*, and proves that *MPKs* play a crucial role in resisting pathogen stress in bananas. In addition, *MPKs* may rely on a large signal transduction network to either directly or indirectly regulate the expression of disease resistance genes to resist pathogen infection. These results also prove the correctness of the KEGG annotations.

On the whole, about half of the *MPKs* were highly expressed under low-temperature stress, and only a few were lowly expressed (Figure 9b). We found that several *MPKs* were highly expressed at each temperature, but also that the expression of some *MPKs* was induced to increase through low temperatures. For example, the expression levels of *MiMPK7*, *MaMPK20,* and *MsMPK15* were the highest at each temperature, while the expression levels of *MiMPK12*, *MaMPK16,* and *MbMPK9* were increased after low temperature induction. Under low-temperature stress, a few genes were differentially expressed from 28 °C to 13 °C. However, the expression of all genes was found to be significantly different from 13 °C to 4 °C, and the situation was also similar from 4 °C to 0 °C. Interestingly, while the expression levels of *MiMPK12*, *MaMPK16,* and *MbMPK9* were upregulated from 28 °C to 4 °C, they were downregulated at 0 °C in the high expression region. *MiMPK15*, *MaMPK10,* and *MsMPK6* were found to be downregulated and upregulated at 0 °C in the low expression region. From 13 °C to 4 °C, the expression levels of all genes at 4 °C were markedly different from 13 °C, which indicates that for the banana plant, the key temperature of low-temperature stress was 4 °C.

### 2.8. The Expression Analysis of Group A under Low-Temperature Stress

**Figure 10:**
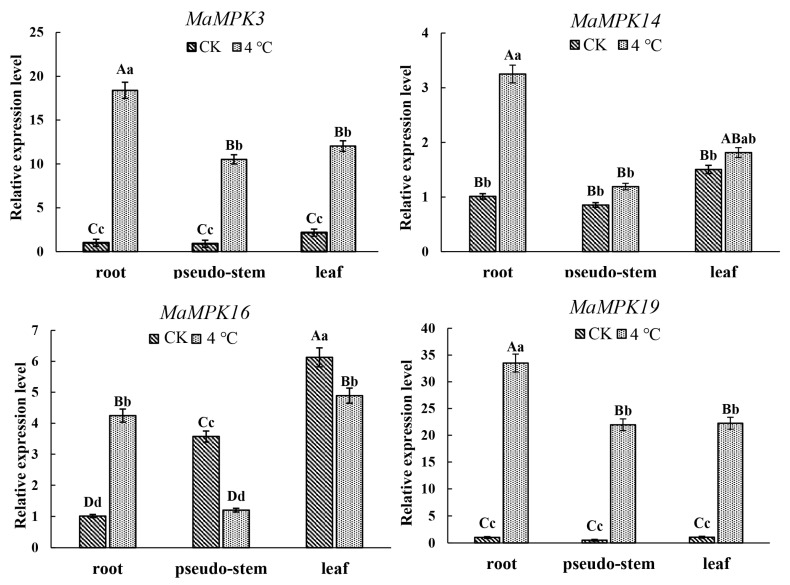
The expression analysis of Group A under low-temperature stress. Uppercase and lowercase letters indicate a significant difference at *p* < 0.01 and 0.05.

**Figure 11:**
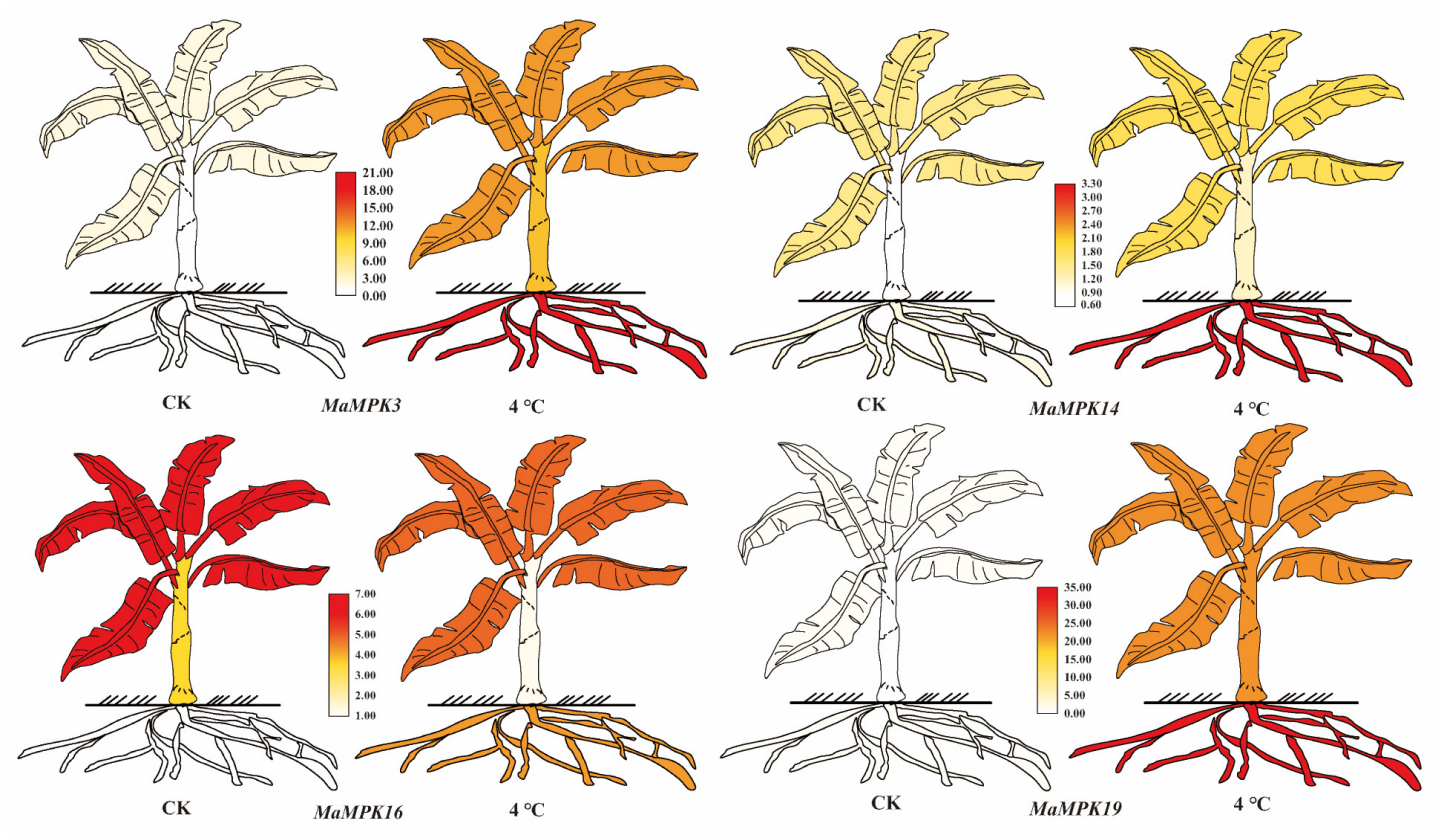
The expression of *MaMPKs* in different tissues. Red represents high expression and white represents low expression. These cartoon heat maps were visualized using TBtools.

Phylogenetic tree, KEGG annotation analysis, and transcriptome results all suggested that Group A may participate in cold resistance, resulting in our focus on *MaMPK3*, *MaMPK14*, *MaMPK16*, and *MaMPK19,* which belong to Group A in *Musa acuminata*. The four genes in the genome of *Musa acuminata* was expressed in the roots, pseudostems, and leaves (Figure 10 and Figure 11). These results showed that the expression levels of *MaMPK3* and *MaMPK19* were upregulated at 4 °C for 24 h. It also indicated that *MaMPK3* and *MaMPK19* were more sensitive to low temperatures. In contrast, the expression of *MaMPK14* did not change significantly in the pseudostems and leaves but was upregulated in the roots. The expression of *MaMPK16* decreased in the pseudostems and leaves while it was increased in the roots. The expression level in the roots was significantly enhanced. The expression levels of *MaMPK3* and *MaMPK19* were more intense compared to the other genes, which were upregulated about 20-fold and 35-fold compared with the control group, respectively. The expression levels of *MaMPK14* and *MaMPK16* in the roots increased about 4-fold. Together, these results indicate that the regulatory mechanisms of *MPKs* under low-temperature stress are diversified in bananas.

## 3. Discussion

### 3.1. Interspecific Specificity and Genome Quality Together Determine the Number of MPKs in Bananas

An interesting phenomenon was that the number of Group B species was associated with the species themselves, while Group A, C, and D were almost unaffected by the species. The gene shrinkage of the Group B subfamily of *Musa balbisiana* and *Musa textilis* was the reason why their *MPKs* were significantly less than *Musa acuminata*, *Musa schizocarpa,* and *Musa itinerans*. Combined with the results of collinearity analysis, *Musa balbisiana* did not display many tandem duplications in Chr04: 3–10 MB, which was different from *Musa acuminata* and *Musa schizocarpa*. The results showed that the number of *MPKs* was contracted when *Musa balbisiana* experienced WGD (whole genome duplication) three times [23,24]. However, since the genomes of *Musa itinerans* and *Musa textilis* have not been assembled to the pseudochromosome level in this study [25,26], they were unable to verify the tandem duplication events in Chr04: 3–10 MB. This inference needs to be proven with the aid of more detailed genomic data of *Musa itinerans* and *Musa textilis*. Therefore, it is also an interesting research direction to further verify the differences of the *MPKs* in bananas through designing genetic complementation experiments for the genes in Chr04: 3–10 MB.

Genome-wide identification results showed that there were differences in the number of *MPKs* in bananas. These differences were closely related to the species, genome sequencing quality, and assembly quality. Five chromosomes achieved a complete telomere-to-telomere assembly of *Musa acuminata* for long-term improvement and optimization of the genome [27,28,29]. A high-quality genome typically indicates that more complete genes have been discovered, which may be the reason why the number of *MPKs* in *Musa acuminata* was found to be more than the others. The *Musa acuminata* and *Musa schizocarpa* genomes were assembled based on nanopore sequencing [30], which had higher continuity and integrity than the *Musa balbisiana* genomes. Overall, the assembly quality of *Musa acuminata*, *Musa balbisiana,* and *Musa schizocarpa* was better than of *Musa itinerans* and *Musa textilis*. According to the current genomic data, the number of *MPKs* is typically determined through interspecific specificity and genome quality. In future studies, more advanced genome sequencing techniques will be used to obtain high-quality genomes. It is an essential subject to completely eliminate the interference of sequencing and assembly techniques, which will not only benefit the comprehension of the influence of interspecific differences on the number of *MPKs* but will also facilitate the bioinformatics analysis and functional verification of the key genes. This will make outstanding contributions to the breeding of bananas using molecular biotechnology.

### 3.2. MPKs Retain the Most Critical Functions during Evolution in the Banana

A large number of studies have shown that Group A and B are involved in abiotic stress and biotic stress [31,32,33]. The number of K14152 pathways is roughly equal across all species of bananas, and all genes of K14152 belong to the Group A subfamily. Notably, the K14152 is closely associated with plant hormone signal transduction and the plant–pathogen interaction. In terms of plant immunity, the resistance of rice blast can be enhanced using the key immune component *OsWRKY31* of the OsMPK cascade [34]. *AtMPK3/6* can be phosphorylated through activating the extracellular ATP receptor P2K1, which improves the defense ability of plants to pathogens [35]. *AtMPK3/6* is involved in the regulation of plant immunity by SUMO (small ubiquitin-related modifier protein) and RNA-binding proteins [36]. In addition, the immune response of mechanosensory trichome cells can be activated through *AtMPK3/6* [37,38].

*MPKs* play a vital role in plant immunity depending on *MAPKKK3/MAPKKK5*-*MKK4/MKK5*-*MPK3/MPK6* [39,40]. The interaction between upstream and downstream genes forms a huge signal transduction network and regulates gene expression. *MPKs* associate with plant hormone synthesis, transport, and signal transduction [41]. The *MKK4/5-MPK6* pathway regulates the polar auxin transport in *Arabidopsis thaliana* [42]. *AtMPK3/6* can phosphorylate ACS in the ethylene synthesis pathway and participate in the process of response to abiotic stress or pathogen infection [43]. *LeMPK1* and *LeMPK2* are involved in the JA synthesis pathway to enhance tomato resistance to pests and diseases [44]. Similarly, *AtMPK3/6* participates in the immune response in the SA signal transduction pathway [45]. The promoter cis-acting elements showed that 76.62%, 25.97%, 88.31%, and 35.06% of the members contained the ABA, Aux, MeJA, and SA response elements, respectively; it indicated that *MaMPKs* were closely related to plant hormones. In conclusion, K14152 is the most conserved entry in *MaMPKs*, and it retains the plant hormone signal transduction pathway and the plant–pathogen interaction pathway under long-term evolution and natural selection. Promoter cis-acting element prediction and transcriptome data can preliminarily prove that *MPKs* play an important role in plant hormone signal transduction and in the plant–pathogen interaction in bananas.

### 3.3. MaMPKs Are Negatively Regulated by miR172 in Low–Temperature Stress

In this study, the expression levels of *MaMPK3*, *MaMPK14*, *MaMPK16*, and *MaMPK19* were significantly upregulated under low-temperature stress. In contrast, mac-miR172a was downregulated, but there was no significant change in the expression levels of mac-miR172b and mac-miR172d. This indicated that mac-miR172a negatively regulates *MaMPKs*. The ICE1-CBF-COR pathway has been widely studied in the cold resistance of bananas. It found that the cold resistance of Dajiao (*Musa* spp. ABB Group) was enhanced after *MpICE1* overexpression, while weakened after RNA interference with *MpMAPK3* [46,47]. It indicated that the MAPK cascade played a positive regulatory role in the cold resistance network of bananas.

A noteworthy phenomenon was that the expression of *MaMPKs* in the leaves was at its highest at 28 °C, and in the roots was highest at 4 °C. The roots were most markedly affected by low temperature. A previous study found that roots improved the ability of the stem cell niche to resist low-temperature stress through auxin accumulation and programmed cell death [48]. *AtMPK6* and its substrate EB1c (end-binding protein 1c) regulated the mitosis of root epidermal cells in *Arabidopsis* [49]. The RGF1–RGI1 ligand–receptor complex regulated downstream PLT1 and PLT2 through YDA-MKK4/5-MPK3/6 and then regulated root meristem development, which has been demonstrated by researchers from different perspectives [50,51]. The stem tip of *Arabidopsis thaliana* converts high-temperature signals into biochemical signals and transmits them to the roots to stimulate the response of the whole plant [52]. From what has been discussed above, *MaMPKs* expression is activated through cold stress and may mainly act on the roots. Therefore, it has been speculated that leaves may transmit low-temperature signals to the roots through the connection between aboveground and underground or the signal transduction pathways. In this way, the roots can provide nutrients or water for the overground to alleviate cold stress.

## 4. Materials and Methods

### 4.1. Plant Materials

The test-tube plantlet of ‘Brazilian’ (*Musa* AAA, Cavendish) was placed at 28 °C for 5 days and were then transplanted into the substrate. The cultural conditions were as follows: temperature 26 ± 2 °C, relative humidity 60–80%, light intensity 2500 Lux, and 12 h light/12 dark. Bananas were moved into the incubator at 4.0 ± 0.5 °C for 24 h, and the other conditions were the same as above. The leaves, pseudostems, and roots were quickly cut into pieces, frozen in liquid nitrogen, and then stored at −80 °C.

### 4.2. Identification of MPK Gene Family in Bananas

The genomic data of *Musa acuminata*, *Musa balbisiana*, *Musa itinerans*, *Musa schizocarpa*, and *Musa textilis* were downloaded from the Banana Genome Database [53] (https://banana-genome-hub.southgreen.fr/content/download, accessed on 15 April 2022). The gene and amino acid sequences of *AtMPKs* were downloaded from the TAIR database (https://www.arabidopsis.org/index.jsp, accessed on 21 April 2022). The candidate gene sequences of *MPKs* were screened using BLAST. The CDD database (https://www.ncbi.nlm.nih.gov/Structure/bwrpsb/bwrpsb.cgi, accessed on 22 April 2022) was used to screen the domains, and the genes containing the STKc_TEY_MAPK and STKc_TDY_MAPK domains were identified as the *MPK* gene family in bananas. Finally, Tbtools [54] was used to visualize the domains.

### 4.3. Protein Physicochemical Properties and Domain Analysis of MPKs

ExPAsy (https://web.expasy.org/protparam/, accessed on 28 April 2022) was used to predict the number of amino acids, molecular weight (Mw), theoretical pI, instability index, aliphatic index, and the grand average of hydropathicity (GRAVY) of the MPK proteins.

### 4.4. Phylogenetic Tree, Collinearity and Chromosome Localization Analysis

The phylogenetic tree containing *Arabidopsis thaliana*, *Musa acuminata*, *Musa balbisiana*, *Musa itinerans*, *Musa schizocarpa*, and *Musa textilis* was constructed through neighbor-joining of MEGA-X and beautified with iTOL (https://itol.embl.de/, accessed on 2 May 2022). Based on the gene location in the genomes, collinearity analysis was shown using Tbtools.

### 4.5. KEGG Annotation Analysis and Promoter Cis-Acting Element Prediction

The protein sequences of *MPKs* were uploaded to KofamKOALA (https://www.genome.jp/tools/kofamkoala/, accessed on 29 August 2022) to predict their KO numbers in KEGG (Kyoto Encyclopedia of Genes and Genomes). The upstream 2000 bp sequences of the CDS were extracted for promoter cis-acting element prediction with Plant CARE (http://bioinformatics.psb.ugent.be/webtools/plantcare/html/, accessed on 29 April 2022).

### 4.6. Expression Analysis of MPKs and Prediction of miRNA

FPKM (fragments per kilobase million) values of *MPKs* were extracted from *foc*TR4 [55] and low temperature [56] transcriptome data, which were displayed using Tbtools. The psRNATarget [57] (https://www.zhaolab.org/psRNATarget/, accessed on 12 November 2022) was used to predict the miRNA that regulates the *MPKs*, and then Cytoscape 3.8.2 was used to draw the interaction network between the miRNAs and genes.

### 4.7. RNA Extraction and qRT-PCR

Total RNA was extracted with the RNAprep Pure Plant Plus Kit (TIANGEN, Beijing, China). The RNA reverse transcription used the 1st strand cDNA synthesis superMix for qPCR (Yeasen, Shanghai, China). The primers for qRT-PCR have been listed in Table S2. The reaction system was as follows: 10 μL qPCR SYBR green master mix (Herui, Fuzhou, China), 0.4 μL forward primer (10 μM), 0.4 μL reverse primer (10 μM), 1 μL cDNA, and 8.2 μL ddH_2_O. The CAC was used as a reference gene. The reverse transcription of miRNAs used the 1st strand cDNA synthesis Kit by stem-loop (Vazyme, Nanjing, China). The sequences of miR172 were derived from NCBI [58] (https://www.ncbi.nlm.nih.gov/geo/query/acc.cgi?acc=GSE77590, accessed on 12 December 2022). The miR172 reverse transcription and qRT-PCR primers have been displayed in Table S2. The reaction system was as follows: 10 μL 2 × miRNA Universal SYBR qPCR master mix (Vazyme, Nanjing, China), 0.4 μL specific primer (10 μM), 0.4 μL mQ primer R (10 μM), 1 μL cDNA, and 8.2 μL ddH_2_O. The qPCR programs were as follows: 95 °C for 5min, 40 cycles of 95 °C for 10 s, and 60 °C for 30 s. The U6 was used as a reference gene. The relative expression levels of *MaMPKs* and miRNAs were calculated using the 2^−ΔΔCt^. SPSS26 was used to analyze the significance test between samples. The instrument used for this experiment was the Roche LightCycler 480.

## Data Availability

Not applicable.

## Supplementary Materials

The following supporting information can be downloaded at: https://www.mdpi.com/article/10.3390/plants12162926/s1, Figure S1: Protein conserved domains of *MPKs* in bananas, Table S1: Basic information and protein physicochemical properties of the *MPK* family in bananas, Table S2: qRT-PCR and reverse transcription primers of *MPKs* and miRNAs, Table S3: annotation of *MPKs* in KEGG.
