# Supplementary figures and images for: Genome-Wide Identification of the MPK Gene Family and Expression Analysis under Low-Temperature Stress in the Banana
Source: Plants (Basel). 2023 Aug 12;12(16):2926. doi: 10.3390/plants12162926 (PMC10460080; doi:10.3390/plants12162926)

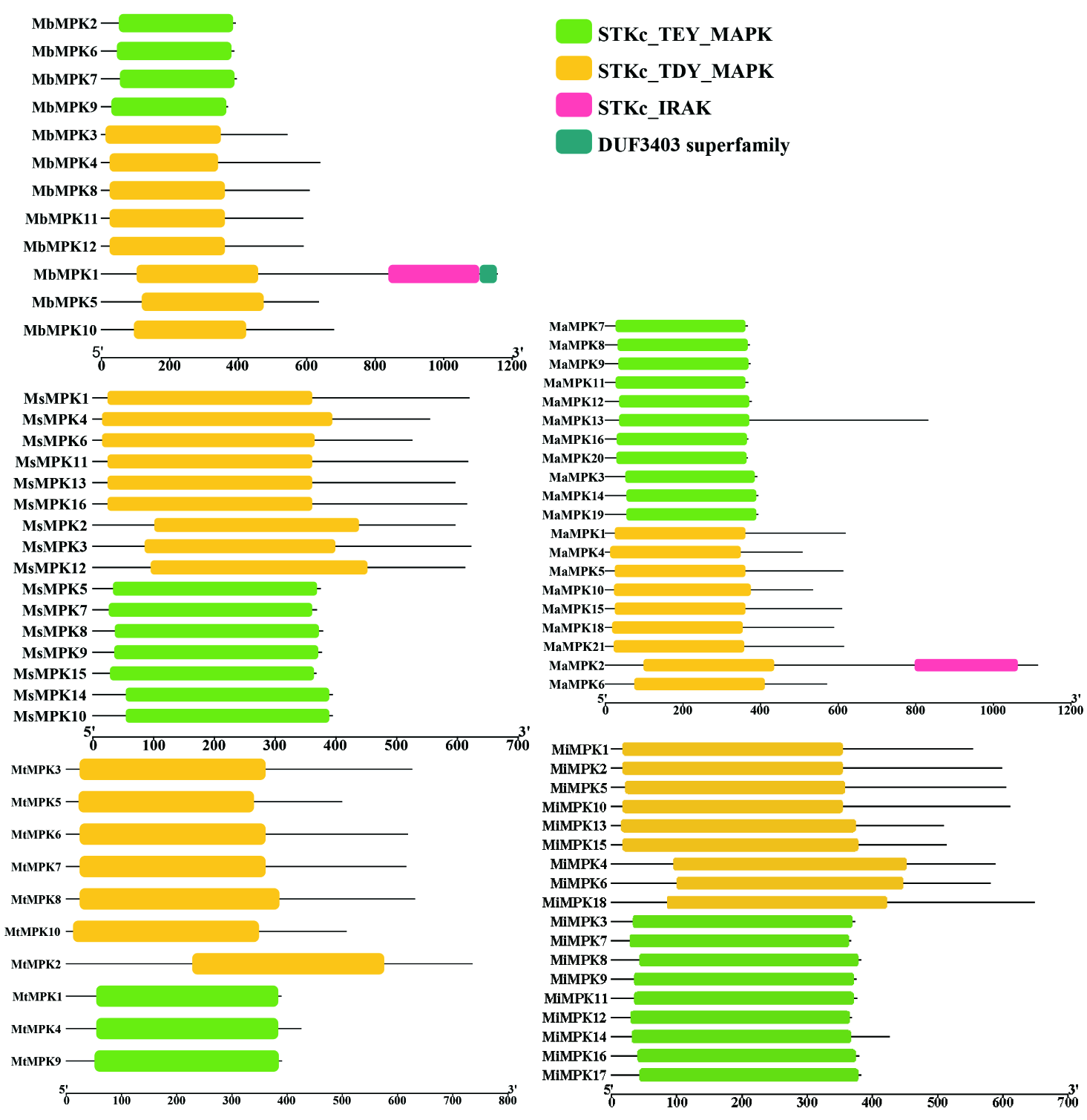

Supplement: Supplementary file 1 [file plants-12-02926-s001.zip › Supplementary Figure S1 Protein conserved domains of MPKs in bananas.tif]
